# The Convention of the American Public Health Association at Chicago

**Published:** 1877-10

**Authors:** 


### THE CONVENTION OF THE AMERICAN PUBLIC HEALTH ASSOCIATION AT CHICAGO.

The Fifth Annual Convention of the American Public Health Association assembled at the Grand Pacific Hotel, on September 25th, 26th and 27th, 1877.

### Tuesday, September 25th—First Day.

The meeting was called to order by Dr. John H. Rauch, of Chicago, president of the association, Dr. Elisha Harris, of New York, officiating as secretary. The president opened the proceedings by introducing Mr. Wirt Dexter, who delivered the address of welcome to the convention. Mr. Dexter spoke of the great importance of the work before the convention, and in fact of any movement having for its object the care of the public health. The absence of disease means wealth and prosperity, and the citizens of Chicago, as shrewd business men, should see this. In point of fact, they are more likely to look with favor on conventions relating to commercial projects than on those which discussed such abstract matters as public health. Men think all men mortal but themselves, but when it can be made to appear that the preservation of public health directly affects the pocket of every trader, small or large, they will see things in their true light.

In conclusion, the members were welcomed to the city, and the kindest wishes expressed for the satisfactory progress of the deliberations.

The next business in order was the address of the president, Dr. John H. Rauch, who read an interesting paper on the geological formation and the sanitary conditions of the city of Chicago. The paper dwelt upon the evident geological changes whereby the lake had receded from its ancient boundaries to its present limits, leaving the present site of the city above the water. The ground on which the city was built was therefore varied in its character, running from hard clay to soft, alluvial mud. These facts must be taken into account if the subject of public health would be fully and intelligently studied. Some of the climatic conditions affecting the general health of Chicago were also pointed out, and the subject of sewerage was touched upon. The paper suggested several directions in which it would be profitable to institute systematic and thorough inquiry, and referred them, with other suggestions, to the action of the convention.

SECRETARY'S REPORT.

The secretary, Dr. Elisha Harris, of New York, then read portions of his annual report. The work marked out by the association was alluded to, and also the difficulties met with in procuring accurate statistics. It advocated the organization of State boards of health, who should pay particular attention to preparing maps which should show not only the ordinary typographical features, but the hydrography and the differences in elevation. By this means only could the influence of swampy and marshy grounds, stagnant waters, and other local causes of mortality be accurately and intelligently studied. This work should be still further and more minutely carried out by local boards in the counties and towns. State boards of health had already been organized in thirteen States, and too much importance could not be attached to the work of these boards if properly carried out. Some of the western States had already made considerable progress in the sanitary work, but much remained undone, and it was for the convention to help along the work.

Mr. E. S. Chesbrough, city engineer, was next in order with a paper on "The Problems, Old and New, in the Sanitary Drainage and Sewerage of Chicago and other Western Cities." Mr. Chesbrough remarked that he was not familar with the systems of many of the western cities, but he would confine himself to the work done in Chicago. He then read an interesting paper, giving a full and complete history of the difficulties met with and overcome in draining the city. The work was traced step by step from the time when the water ran in the middle of the street, making bottomless mud-puddles in the principal streets, down to the present complete system of tunnels and sewers which seemed to meet all the requirements. Mr. Chesbrongh explained the system by the aid of a map, and answered a number of questions by the members.

The order of business was changed, somewhat, and Mr. Charles F. Folsom, secretary of the State board of health of Massachusetts, spoke at considerable length, and read the draft of a law on "Pollution of Streams." Mr. Folsom spoke of the absolute necessity of some legislation of this character, especially in some of the eastern States where, in some cases, the water in the streams was so foul that the manufactories found it necessary to use a filter before the water could be available for their business. The effect of these pollutions on the health of the neighborhood was fully explained. The subject of pollution of streams, and other sources from which supplies of drinking water were derived for cities and towns, was discussed at length, and Mr. Folsom read the draft of a proposed law for the State of Massachusetts. The law enacted heavy penalties for any act tending to render the water impure or unfit for use. It also gave large discretionary powers to the State boards of health to investigate and prosecute all offenders.

The bill was the subject of much discussion, participated in by Dr. Bevan, Prof. Lyman, Dr. Harris, and others, and on the whole was favorably received. On motion the proposed bill was referred to a special committee of five for some revision and amendments.

The report of the treasurer of the association was received, showing a paid-up membership of two hundred and sixty. No objection being raised, the report was referred to the executive committee.

The secretary reported a list of about one hundred names which had been proposed as honorary members and favorably acted upon by the executive committee. On motion, they were formally elected members.

The meeting adjourned.

THE EVENING SESSION

commenced in the ladies' ordinary at 8 o'clock, Dr. N. S. Davis presiding. Dr. Davis made a few remarks on the early history of Chicago, and pleasant reminiscences of the times when there was "no bottom" on Lake street, and the bull-frogs were the only occupants of the site of the Grand Pacific.

Dr. Hosmer A. Johnson then read a paper on "The Sanitary Geography of Phthisis Pulmonalis and other Pulmonary Diseases in Chicago and Other Cities of the Northwest." The questions discussed were :

1. What is the relative frequency of phthisis pulmonalis in the north-western States as compared with the north-eastern States of the Union?

2. Does phthisis pulmonalis, relatively to other causes of death, become more frequent as population increases in the United States?

3. What is the ratio of increase, if any, in the two sections of the country mentioned?

4. What are the causes that modify the prevalence of this disease in the two sections, and more especially in the north-western states?

These questions were discussed at length, and an imposing array of figures and statistics adduced. The conclusions arrived at seemed to be not a little uncertain to the author himself, particularly as there were so many modifying causes and local influences, that no general rule could be laid down.

Dr. Turner made a few remarks on the subject, and was followed by Dr. Gihon, Medical Inspector, United States Navy, who gave the result of his observations. He said that it was an acknowledged fact that lung diseases were much more prevalent in what is known as "wet" vessels than in "dry" ones. The medical officers had waged a constant warfare against the practice of washing the decks too frequently. In leaky vessels, or vessels on which decks were kept constantly damp by the over-cleanliness of martinet officers, there was always an excess of consumption and lung diseases; while on vessels with perfectly dry decks these diseases were comparatively rare. The rule seemed to be that a prevailing moist atmosphere, wherever found, was conducive to this class of complaints.

"The Relation of Hygiene to Higher Education" was the subject of an interesting and able paper by Dr. J. M. Gregory, president of the Industrial University of Illinois. The ground was taken that the preservation of health was even of more importance in the colleges and universities than in the primary schools. The necessity of pure air, plenty of exercise, and the danger of a forcing process on the bodily and mental organization of the student were fully set forth.

The meeting then adjourned.

WEDNESDAY, SEPTEMBER 26TH. SECOND DAY.

The second day's session opened in the ladies' ordinary of the Grand Pacific, the president, Dr. John H. Rauch, in the chair. The first paper read was by Dr. J. L. Andrew, of La Porte, Indiana. The author discussed the influence of trees in condensing moisture from the air and retaining it in the ground. Examples were multiplied to prove the fact that a general clearing out of the forests invariably produced a diminution in the rainfall. Probably the best example was the condition of things in northern Africa, where, by the boring of artesian wells, and planting of trees, stations and oases had been established in the great Sahara desert, and these spots were now frequently visited by showers. A still stronger proof was the fact that the discovered sources of the Nile, which sends its immense floods over the lower country, were in the dense forests and jungles under the equator; jungles so dense that the light of the sun could scarcely penetrate. Similar examples of the value of tree-planting could be found on our western plains, where the testimony of army officers was unanimously in favor of the good results from such artificial forests. The paper also discussed the value of timber belts and undergrowth in counteracting the malarial effects of swampy and marshy ground, the conclusion being that such timber growths offered a great protection from this class of poisons. The eucalyptus among trees and the sunflower among plants were mentioned, as being particularly valuable for neutralizing and destroying the malarial poisons in exposed situations. The author recommended a still further study of this subject, and expressed the belief that the science of prevention of disease was yet in its infancy. He argued that all regions unfit for profitable cultivation should be devoted to forests, and these forests should be protected by an enlightened public sentiment as well as by legal enactments, on the ground that it was a sanitary as well as an economical necessity.

The secretary then presented a paper by Prof. Brewer, of the Sheffield scientific school of Yale college, on the subject of tree-planting, the arguments and conclusions being similar to those of the preceding paper. It carried the inquiry still further by giving some results of experiments with different species of trees with a view to determining the sanitary qualities of each. The paper was supplemented by some remarks by the secretary, who instanced, the work of laying out a town on Hampstead plains, on Long Island, by A. T. Stewart. In this case the streets were laid out and trees planted in advance of any other improvements.

Dr. Churchill, of Galesburg, spoke of the changes noted in that climate since it was settled. A marked decrease in the rain-fall had resulted, or, at least, followed the clearing away of the forests.

The next paper was by Dr. Ezra M. Hunt, secretary of the State board of health of New Jersey, the subject being, "A Memorandum of Observations and Propositions concerning the Sanitation of Individuals, with Reference to the Arrest and Prevention of Infectious Maladies." In the absence of the author, the secretary read the principal portion of it, accompanied with explanatory remarks of his own. The paper was in substance the result of the observations and practice of the author in reference to individual cases with which he had to deal.

A paper, "A Review of the Teachings of Twenty-two Years' Record of the Mortality from Croup, Diphtheria, and Scarlatina in a City," by Dr. Edwin M. Snow, superintendent of public health, Providence, R. I., was also read by the secretary, and consisted principally of carefully prepared statistics of the mortality resulting from these diseases in the city of Providence. Both these papers were accepted, on motion, and ordered printed with the proceedings of the association.

The secretary read a letter from Dr. Samuel Choppan, president of the Louisiana board of health, regretting the inability of that gentleman to be present at the meeting. The letter stated that the arrival of several ships from Havana, with cases of yellow fever on board, had demanded the writer's whole time and attention. He proposed, however, to prepare a paper on the subject of yellow fever, with reference to its prevention by quarantine regulations.

Dr. Henry M. Lyman, of Chicago, read a paper on "'Stamping out' Scarlatina, and the Extinguishment of Zymotic Disease." He first called attention to a chart, showing the comparative amount of mortality from scarlet fever in Chicago since 1850. The chart showed that there had been a scarlet fever epidemic on an average of every five years, and that each visitation had lasted about two years. The death-rate was higher in extremes of heat or cold, the mortality being greater in the midsummer or the midwinter months. The paper suggested measures looking to a prevention of the disease, and took strong grounds against the displaying of the obnoxious cards on houses visited by the disease. It was impossible to isolate cases in a great city like Chicago; and the people in general revolted against the yellow-card nuisance.

The result of the law compelling the display of cards, was that not less than half the cases were not reported. The family either did not call in a physician, or called in some quack who would not report the case. In consequence of this course the disease was still further spread and the mortality increasing. The cost of the cards and tacks would pnblish, in pamphlet-form, such information as would vastly reduce the danger. The masses should be enlightened and their prejudices overcome, rather than attempts be made to coerce or drive them to certain useless measures. Other strictures were made on the conduct of the health officials in general.

Dr. Folsom, at the close of the reading, deprecated such discussions as the paper must necessarily lead to, on the ground that it was running too much to personalities. He did not think the paper a proper one for the association to spend time listening to, and hoped that in future the executive committee would look over the papers, and, by suppressing all matters relating to local troubles, encourage a higher order of discussion.

Dr. Kearney, health officer of Cincinnati, said a few words in a similar strain, and hoped there would be no discussion.

AFTERNOON SESSION.

Dr. Azel Ames, Jr., of Boston, on "The Removal and Utilization of Domestic Excreta." The writer held that out of all the methods proposed for removing the human excreta, none had paid sufficient attention to the utilization of the product. The questions to be met were the speedy, inoffensive, and economical removal of the human excreta, and the best means of rendering it profitable. The different systems were discussed, as the vault system, the water-closet system, the pail system, and the pneumatic system, each having its advocates. The vault system, as used in isolated houses and country towns, was offensive and in many cases dangerous. The earth closet system offered many advantages, but could hardly be made to work in large cities. The water system was perfect as far as inoffensive and speedy removal was concerned, but, unless by irrigation, it was impossible to use the sewerage. The pail system was in use in China, and consisted in the daily removal of the excreta, but was expensive, offensive, and open to many objections. The pneumatic system, as first made use of by Capt. Charles Liernur, consisted in theory of a tank at a street corner or other convenient place, connected by tubes with the closets in the several houses. By means of a portable engine a vacuum is formed in the tank, the consequent pressure drawing the substance from the house to the tank, from which it is then removed and utilized. This system was further improved by the inventor, by connecting the street corner tanks with a larger tank at the central works. By pumping the air from the tank a vacuum was formed, and this in turn forming vacuums in the smaller tanks, the result was that the excreta were thus collected by pneumatic pressure. From the main tank the substance was treated with sulphuric acid or some other deodorizer, and made to pass over a drum heated by steam and revolving in a superheated atmosphere. This reduced the substance to a powder, when it was ready for shipment in barrels. This system had been found practicable, and was in successful operation in Amsterdam, and one or two other European cities, the prepared powder being found of sufficient value to pay all the running expenses, and provide a sinking fund toward the repayment of the first cost. This method in successful operation seemed to meet all the requirements, for a perfect system. The question to be met at present was how to render least offensive and least objectionable the vault system, as this is the one in almost general use outside of large cities. The ordinary methods of cleaning the privy vaults by night scavengers was too disagreeable and disgusting to be tolerated. To meet this objection a number of patent appliances had been introduced, all of which were discussed by the author. Some of them, he said, were positively dangerous, and in some instances had exploded in the attempt to confine the gases. A machine similar in the operating principle to the Liernur pneumatic process, seemed to meet the approval of the author. In conclusion, he held that no system could be called a success unless it provided for a utilization of the excreta, and their return to the soil in the shape of a fertilizer.

Dr. N. S. Davis had prepared a paper on "The Means of Diminishing Infant Mortality from Bowel Affections." The doctor spoke of the causes of bowel affections, which, in gen-

6

eral terms, might be stated as the result of the high temperature of the hot summer nights. It might also be affected by the presence or absence of ozone, or by electrical conditions, but he believed the principal cause to be the hot nights and the absence of proper ventilation. A high temperature during the day was not so productive of complaints as the continuous heat during the night. It was well known that nearly all bowel affections, including cholera, first affected the patient in the hours between midnight and morning. The heat being the cause, what should be done by way of remedy? Floating hospitals and country air were well enough in their way, but all could not avail themselves of these advantages. The next best thing to be done was to secure all the ventilation possible and, by a cool bath before putting the child to bed, to reduce, the temperature of the body and quiet the nerves of the bowels. A wet towel around the child's body was also in many cases productive of a great deal of good. By proper attention to these simple appliances, during the prevalence of a heated term, the infant mortality may be reduced at least one-half.

Dr. Hamill, physician in charge of the Floating hospital, gave his testimony to corroborate the positions taken by Dr. Davis. He had seen children who on shore were oppressed and almost exhausted, revive even on the trip from the shore to the hospital boat, and on reaching the boat sink into a quiet and refreshing slumber.

Dr. Lyman's experience was similar, and he considered that a little attention to the cause, and some simple precautions like those suggested by Dr. Davis, would be of great benefit to the children.

In answer to a question by Dr. E. W. Gray as to observations on the temperature of the blood, Dr. Davis again spoke of the effect of heat on the system. When the air is so heated and rarefied, the child's lungs are too small to take a sufficient quantity to supply the body with oxygen.

The child, therefore, is soon prostrated with exhaustion, and a relaxation of the nerve centres ensues, acting most strongly on the bowels.

Dr. Turner said that children were largely fed on artificial food and not the mother's milk. When cow's milk was used it was sometimes watered, and other substances were added to it with a view of rendering it more like the natural food. Indeed he had met with numerous cases where the child actually died of starvation. He had always advocated plenty of pure cow's milk, and met with good results.

Several other members gave expression to their views, but nothing additional was elicited.

"A Report and Discussion upon the Experience of the Department of Physical Education and Hygiene of Amherst College," was the subject of a paper by Prof. E. Hitchcock, of Amherst, Mass., and was read, in the absence of the author, by Mr. John Woodbridge. The paper was a detailed description of the buildings and apparatus in use in Amherst college, with some observations as to the results of the system of physical education on the students. The department is in charge of a professor, and a regular routine of exercises is carried out, the attendance of the students being made obligatory. The description was tedious and uninteresting to any but an Amherst man, and had the effect of rapidly thinning out the audience.

Dr. Coan, of Quincy, read a short paper on the relative amount of physical culture required for male and female students. The doctor seemed to think that the girls were fully as capable, physically, of pursuing a college course as the boys.

The meeting adjourned.

EVENING SESSION.

The meeting was called to order at 8 o'clock, Dr. Ingals, of Chicago, presiding. The first paper was "A Statement and Discussion of the Sanitary and Economical Importance of the best Medical and Surgical Treatment of the Needy Poor," by Dr. Edmund Andrews, of Chicago. The doctor argued in favor of prompt and efficient surgical or medical aid to the poor, on the ground that it was much more economical for society to furnish a surgeon, and even mechanical appliances in case of accident, than to have the unfortunate man a permanent charge on the public as a pauper. He held that the cost of supporting him as a pauper was not to be reckoned in comparison with the loss of the man himself to society. He instanced cases of rupture, for example, where a trifling expense would furnish a truss, whereby the man could return to his accustomed work and not be obliged to spend the balance of his life as a helpless pauper. Other examples of a similar character were given, showing what an extent of suffering and misery could be prevented by a well-directed public movement to aid men who meet with serious accidents while at their work, and who are utterly unable to secure the proper treatment for themselves. It was a disgrace, he said, that deformed and crippled creatures should be allowed to walk the streets when a proper treatment in the beginning would have restored them to health and usefulness.

Dr. Chancellor, of the State board of health of Maryland, was called on for his views, and said that he was strongly in favor not only of surgical treatment, but also of medical aid in case of sickness in their families. He had given particular attention to this subject, and had made reports on the condition of some of the charitable institutions in his own State. The workingman was often driven to ask for aid by sickness or death in his family. The feeling of shame was thus overcome, and he was liable to become a voluntary pauper. By furnishing medical aid at, perhaps, a nominal cost, and so preserving the feeling of independence, the man would be truly benefited and society be the gainer.

Dr. Harris, the secretary, made some remarks on the same subject, and offered the following:

*Whereas*, Public attention is being urgently invited to the duty of adopting measures for repressing and preventing pauperism; and,

*Whereas*, There are causes of bodily disabilities which induce dependence and the entailment of pauperism, resulting from the neglect and incompetence of the attendance and medical sanitary care received by the needy poor when such care should be given:

*Resolved*, That in the judgment of this association, the official inspection and inquiry for ascertaining and repressing the cause of bodily disabilities and pauperism, is an important public service in the interests of hygiene and the public welfare, and that those interested require that this duty should be continued with faithfulness, and the results be published widely for the information of the public.

The next paper was by Dr. Henry M. Lyman, of Chicago, on "The Present State of Exact Knowledge of Causation and Prevention of Epidemic Diseases." The doctor first called attention to the difference between poisons and infectious substances. Snake bites were examples of poisons, while the bite of a rabid dog was an illustration of the latter. The real difference between a poison and an infection-virus was that in the case of poisoning the effect was dependent upon the amount of the matter injected, it having no power of reproduction, while a virus was endowed with the power of self-multiplication, and soon extended over the whole body. Hydrocyanic acid, nettle juice, and the venom of a copperhead, may be accepted as types of poison, while the fluids of the dissecting-room, the lymph of a vaccine vesicle, and the exhalations of measles, furnish illustrations of the nature of an infectious substance. By one or the other of these modes nearly all epidemic diseases are communicated. The paper gave numerous illustrations explaining his meaning, and showing how the different infectious diseases may be communicated. The action of the infecting substance, in the same classes of epidemics, is not as yet fully understood, and was a topic calling for careful study and investigation. In some cases a family, or even a community, may be so inoculated with poisonous virus of a milder form, as to be predisposed to the ravages of a dangerous epidemic. The paper treated at length of the different epidemic diseases in detail, showing how mistakes were liable to arise in looking for the true causes of the spread of the contagion.

Rev. Brooke Herford then delivered an address on the subject of "Public Health and Public Holidays." He said that some of the English writers had complained that life in England was run on a high pressure. If this was true in England, how much more could it be said of this country.

The business in this country was run at a racing speed, and was a terrible strain on the mind and body. No rest was allowed night or day. Men were at their desks at seven o'clock in the morning and remained till after midnight. It used to be said that every French soldier carried a field-marshal's baton in his knapsack; and it might be said that every clerk or office boy saw himself in the future a Tweed or a Stewart. Men by such constant application lost all taste for leisure, and could do nothing but drive along. They will tell you that they cannot possibly leave their business; and in a few years a broken-down, prematurely-old man, who trembles at every noise, is all that is left of the once vigorous worker. In England the case was different. Holidays are far more numerous, and are better observed. Easter-time, Good-Friday, Whitsuntide, besides the week's holiday at Christmas-time, were universally observed, to say nothing of the Queen's birthday and anniversary days. In this country there were hardly four holidays observed in the year, and these only partially. It has been said that an American with a holiday is the most miserable being on earth. This was in a measure true. The business man had no idea of spending time for culture, and wanted to carry his business into everything. The Englishman, on the contrary, seized upon every possible occasion for the laying aside of his business cares and giving himself up to pleasure.

The speaker pointed out the inevitable results to the health and strength, of these two courses, showing the advantage strongly in favor of the Englishman. In conclusion, he spoke of the movement in England for a Saturday half-holiday, which had succeeded in the busy city of Manchester and other English towns, and expressed a hope that this movement would be taken up here and carried through to a similar success.

The address, which was an eloquent and interesting one, was received with frequent applause. A vote of thanks was tendered for the paper, and it was ordered to be p inted with the proceedings of the Association.

### Thursday, September 27th—Third Day.

#### MORNING SESSION.

The meeting was opened by Dr. DeWolf, Health Commissioner of Chicago, with a paper on the "Destruction of Offensive Gases from Rendering Tanks and Fertilizing Establishments." The doctor spoke of the importance and the difficulty of meeting this problem. There were three hundred thousand beeves and three million two hundred thousand hogs slaughtered in and around this city last year. Each beef produced from fifty to sixty pounds of offal, and each hog, from twenty to thirty pounds. This gave some idea of the magnitude of the nuisance-producing business. He then gave a description of the rendering-tanks in which every thing of an offal nature was collected and subjected to a steam pressure. It was the gases from these tanks which caused the trouble. There were two hundred and forty-two of these rendering vats, and the gases were formerly all allowed to escape into the open air. The various means proposed for condensing and destroying these gases were explained at length.

He spoke of the catch-basin which had been introduced, by which about twenty tons of solid animal matter, which was formerly conveyed into the river through the sewers, was now collected and utilized. He referred, in closing, to the recent scarlet fever epidemic, and of the attempts made to arrest and quarantine the disease, as well as the probable effect of the poisonous gases alluded to, on the sanitary condition of the city.

Dr. Folsom, of Boston, made some remarks on the subject of the paper, and thought that it was absolutely necessary to subject the offal to the condensing process while it was perfectly fresh. He also spoke of the scarlet fever question, and this led to remarks by other gentlemen present, Dr. DeWolf making the statement that the number of scarlet fever cases had been as great in the best wards as in the worst, as shown by the reports of the Board of Health, but that the death rate was materially modified by the good sanitary condition of the wards.

Dr. Johnson agreed with the last speaker. His experience was that the local sanitary conditions had little or no effect in preventing the spread of the disease, although it certainly had an influence on the rate of mortality. Considering all the difficulties to be encountered, the city had reason to congratulate itself on its sanitary condition. The attempt to regulate the stench-producing establishments on the southwest border of the city, had been found almost impossible. People living in the immediate neighborhoods of these places, would come forward and swear that they were the most healthy places in the world. The doctor accounted for this on the principle that the internal conditions of the body adapted themselves to the external conditions. When the dry southwest winds came over these places, they gathered up the gases and carried them over the city until a cooler, moist current of air was encountered near the lake, and the poisonous gases deposited on the inhabitants. The warfare agaiust these stench-producing establishments must be kept up until the evil was overcome.

This closed the discussion, and the auditing committee reported that the affairs of the treasurer and secretary had been examined and the reports of those officers found to be correct.

The election of officers for the ensuing year, which followed, resulted as follows: President. Dr. Elisha Harris, Health Commissioner of New York; First Vice-President, Dr. J. L. Cabell, President State Board of Health, University of Virginia, Charlotteville; Second Vice-President, Dr. Hosmer A. Johnson, Chicago; Secretary, Dr. R. H. James, Secretary New York Board of Health; Treasurer, Dr. Charles F. Folsom, Secretary State Board of Health, Boston, Mass.; Executive Committee, Dr. John H. Rauch, President State Board of Health, Chicago, Ill.; Dr. C. B. White, ex-President State Board of Health, New Orleans, La.; Dr. C. W. Chancellor, Secretary State Board of Health, Maryland; Dr. J. T. Reeve, Secretary State Board of Health, Appleton, Wis.; Dr. C. N. Hewitt, Secretary State Board of Health, Red Wing, Minn.; Dr. Thomas J. Turner, Medical Inspector United States Navy, Washington, D. C.

AFTERNOON SESSION.

The first paper read was by Dr. Elisha Harris, of New York, on "The Statement on the Outlines of a Plan for Securing Uniformity and Completeness in the Registration of Vital Statistics and Prevalent Diseases."

The paper gave some of the methods in use in New York, Boston, and other of the eastern cities. The object of the paper was to prove the necessity of some national movement looking to a complete compilation and record of all facts bearing on the subject of vital statistics. On motion of Dr. Hewitt, the subject matter and the recommendation of the paper were referred to the several boards of health.

The Secretary then called attention to a number of papers in his hand on different subjects, which there was not time to read. All of these papers were referred to the publishing committee.

Dr. Charles N. Hewitt, Secretary of the State Board of Health, Minnesota, read an interesting paper on "The Relation of Hygiene to Lower Education." The paper took the ground that a study of the principles of hygiene was worthy of more attention in the common schools than the study of physiology as ordinarily pursued. A child should be taught what to do in cases of sudden sickness or accidents, and how to preserve health rather than have his brains crammed with the physiological names of the different bones and tissues of the body. The lower classes must be reached, and for this end the family physician must lend his aid by personal advice and the distribution of small tracts on hygienic topics.

The newspapers should also be made use of to disseminate the information, and a good work could be done in the Sunday schools. He also urged the value for this purpose of suitable text-books, which should be prepared for use in the common schools, instead of the worthless physiologies.

The Secretary then mentioned the fact that an association like to the present was in session in Nuremburg, Germany. The order of business laid out for the German fellow-workers was read, the programme extending over three days. An invitation for an exchange of publications was accepted on motion of the Secretary.

Dr. Chancellor moved that the customary thanks be extended to the proprietors of the Grand Pacific, the newspapers, Mayor Heath, and the citizens of Chicago in general, for kindness and courtesy shown to the Association.

The time and place for holding the next annual convention were referred to the executive committee, with the recommendation that the meeting be held a little earlier in the season.

The resignation of Dr. Charles F. Folsom, treasurer elect, was accepted at his earnest request, and Dr. H. B. Baker, Secretary of the State Board of Health, Lansing, Mich., was elected to the position.

After a vote of thanks to the retiring officers of the Association, and some remarks by the Secretary, the convention was declared adjourned *sine die*.

The next meeting of the Association will be held at White Sulphur Springs, Va., during next August.

